# Transcranial Magnetic Stimulation in Obsessive-Compulsive Disorder and Adolescent Depression: A Systematic Review of Efficacy, Safety, and Predictors of Treatment Response

**DOI:** 10.7759/cureus.92496

**Published:** 2025-09-16

**Authors:** Naga Venkata Satish Babu Bodapati

**Affiliations:** 1 Psychiatry and Behavioral Sciences, Sunshine Behavioral Health Services Inc., Bakersfield, USA

**Keywords:** adolescent depression, neuromodulation, obsessive-compulsive disorder, ocd, systematic review, tms, transcranial magnetic stimulation, treatment predictors

## Abstract

Obsessive-compulsive disorder (OCD) and adolescent depression are debilitating conditions where standard treatments often yield suboptimal outcomes. Transcranial magnetic stimulation (TMS) is a non-invasive neuromodulation technique with established efficacy in adults with OCD, but its role in adolescent depression remains less defined. This systematic review aims to synthesize the current evidence on the efficacy, safety, and predictors of treatment response for TMS in adults with OCD and adolescents with depression. This review was conducted in accordance with the PRISMA (Preferred Reporting Items for Systematic Reviews and Meta-Analyses) guidelines. A systematic search of PubMed/MEDLINE, Embase, PsycINFO, Web of Science, IEEE Xplore, and ClinicalTrials.gov was performed for studies published between 2020 and 2025. Eligible studies included randomized controlled trials (RCTs) and non-randomized interventional studies of TMS in adults with OCD or adolescents with depression. Data on efficacy, safety, and predictors of response were extracted. Risk of bias was assessed using the Cochrane RoB 2 and ROBINS-I tools. Nine studies were included (six on OCD and three on adolescent depression). For OCD, four of six RCTs reported significant reductions in Y-BOCS scores with active TMS compared to sham, targeting regions such as the dorsal anterior cingulate cortex (dACC), orbitofrontal cortex (OFC), and supplementary motor area (SMA). Protocols were highly heterogeneous, including accelerated theta-burst stimulation. For adolescent depression, one RCT combining repetitive TMS (rTMS) with fluoxetine in first-episode patients showed very high response rates compared to sham. Two open-label studies in treatment-resistant depression reported symptom reduction and correlated neural changes, though they lacked control groups. TMS was generally well-tolerated, with mostly mild adverse events; one serious event was reported. Neurocognitive testing showed no negative effects. The investigation of predictors of response was notably limited, with only preliminary evidence suggesting roles for neurocognitive performance, neural activation patterns, and symptom subtype. TMS demonstrates promise as an effective and safe intervention for both adults with OCD and adolescents with depression, though the evidence base is still evolving. Significant variability in protocols and a lack of long-term follow-up data exist. The most critical gap is the absence of robust predictors to guide personalized treatment selection. Future research should prioritize large, rigorous trials that focus on identifying biomarkers and clinical factors predictive of responses to optimize TMS therapy for these populations.

## Introduction and background

Adolescent depression and obsessive-compulsive disorder (OCD) represent two of the most prevalent and debilitating psychiatric conditions, often leading to significant impairment in academic, social, and emotional development [1]. Depression during adolescence is typically characterized by persistent sadness, irritability, loss of interest or pleasure, fatigue, feelings of worthlessness, difficulties in concentration, and suicidal ideation, with many adolescents also experiencing functional impairment in school and relationships [2]. It is associated with a heightened risk of recurrent episodes, suicidality, and long-term psychosocial difficulties. In contrast, OCD in adults is characterized by intrusive and persistent thoughts (obsessions) and repetitive behaviors or mental acts (compulsions) that are performed to reduce distress, often severely disrupting daily functioning [2]. Despite the availability of pharmacological and psychotherapeutic interventions, treatment response in both conditions remains suboptimal, with a substantial proportion of patients experiencing only partial relief or intolerable side effects [3]. These challenges underscore the urgent need for safe and effective alternative treatment strategies.

Transcranial magnetic stimulation (TMS), a non-invasive neuromodulation technique, has emerged as a promising intervention in psychiatry [4]. By delivering magnetic pulses to specific cortical regions, TMS modulates neural activity in brain networks implicated in mood regulation and compulsivity, including the dorsolateral prefrontal cortex and supplementary motor areas (SMAs) [5]. While TMS has gained regulatory approval for the treatment of major depressive disorder in adults and has demonstrated efficacy in adults with OCD, its application in adolescents with depression remains an area of growing but still limited research [6]. Importantly, adolescence represents a critical developmental window during which brain circuits involved in emotional regulation and cognitive control are still maturing, raising both opportunities and challenges for neuromodulation-based interventions [7].

Existing studies investigating TMS in adults with OCD and in adolescents with depression suggest potential benefits in terms of symptom reduction, functional improvement, and tolerability [7]. However, the evidence base is still fragmented, with variability in stimulation protocols, sample characteristics, and outcome measures. Moreover, concerns remain regarding the long-term safety of repeated stimulation in developing brains, as well as the identification of reliable predictors of treatment response that could inform patient selection and optimize clinical outcomes.

Given these gaps, a systematic review of TMS in adults with OCD and adolescents with depression is warranted to synthesize the current state of evidence. This review aims to evaluate the efficacy and safety of TMS in these populations and to explore predictors of treatment response, thereby providing insights that may guide future clinical practice and research directions.

## Review

Methodology

Protocol and Registration

This systematic review was conducted in accordance with the Preferred Reporting Items for Systematic Reviews and Meta-Analyses (PRISMA) guidelines [8]. The review protocol was developed in advance to ensure transparency and methodological rigor. Although not prospectively registered on PROSPERO due to time constraints, the design and reporting strictly adhered to PRISMA recommendations.

Eligibility Criteria

Studies were selected based on predefined eligibility criteria to ensure consistency and relevance. Only original research articles were included, while reviews, editorials, and commentaries were excluded. The time frame was restricted to publications from January 2020 to August 2025 in order to capture the most recent and clinically relevant evidence on TMS in adolescent populations with depression or adults with OCD. Both randomized controlled trials (RCTs) and non-randomized interventional studies were eligible. Case reports and series with fewer than five participants were excluded due to limited generalizability.

The detailed inclusion and exclusion criteria are summarized in Table 1.

**Table 1:** Eligibility Criteria TMS: transcranial magnetic stimulation; rTMS: repetitive transcranial magnetic stimulation; iTBS: intermittent theta-burst stimulation; OCD: obsessive-compulsive disorder; DSM-5: Diagnostic and Statistical Manual of Mental Disorders, Fifth Edition; ICD-10: International Statistical Classification of Diseases and Related Health Problems, Tenth Revision; RCTs: randomized controlled trials; ECT: electroconvulsive therapy; tDCS: transcranial direct current stimulation; DBS: deep brain stimulation

Category	Inclusion Criteria	Exclusion Criteria
Population	Adolescents (10–19 years) diagnosed with depression or adults OCD using standardized diagnostic criteria (DSM-5, ICD-10, or equivalent)	Adults with depression (>19 years), children (<10 years), or mixed samples without stratified adolescent data
Intervention	Transcranial Magnetic Stimulation (any type: rTMS, iTBS, deep TMS, etc.)	Other neuromodulation interventions (ECT, tDCS, DBS, etc.)
Comparator	Placebo/sham, treatment as usual, or pharmacological/psychotherapeutic comparators	None
Outcomes	Efficacy (response, remission, symptom reduction), safety/tolerability (adverse events, dropouts), predictors of response	Studies not reporting clinical outcomes (e.g., technical or mechanistic only)
Study Design	RCTs, non-randomized interventional studies, and cohort studies	Case reports, reviews, editorials, conference abstracts without full data
Time Frame	2020–2025	Prior to 2020
Language	English	Non-English without available translation

Information Sources

Electronic databases were systematically searched to identify relevant studies. The following databases were included: PubMed/MEDLINE, Embase, PsycINFO, Web of Science, and IEEE Xplore. To supplement this, trial registries such as ClinicalTrials.gov were searched for ongoing or unpublished studies. Additionally, reference lists of eligible articles and cited sources were screened to ensure completeness of the evidence base. The last search was conducted in August 2025.

Search Strategy

A comprehensive search strategy was designed using a combination of Medical Subject Headings (MeSH) and free-text terms relating to "transcranial magnetic stimulation", "adolescents", "depression", and "obsessive-compulsive disorder". Boolean operators were applied to ensure sensitivity and specificity. An example of the PubMed search strategy is presented in Table 2. The same core structure was adapted to other databases with necessary modifications.

**Table 2:** Example Search Strategy (PubMed/MEDLINE)

Search Set	Search Terms
#1	"Transcranial Magnetic Stimulation"[MeSH] OR "TMS" OR "rTMS" OR "iTBS" OR "deep TMS"
#2	"Adolescent"[MeSH] OR "Teen" OR "Youth" OR "Young people"
#3	"Depression"[MeSH] OR "Major Depressive Disorder" OR "MDD"
#4	"Obsessive-Compulsive Disorder"[MeSH] OR "OCD"
#5	#1 AND #2 AND (#3 OR #4)
Limits	Publication date 2020–2025; English language

Study Selection

All records retrieved from the databases were imported into reference management software, and duplicates were removed. The titles and abstracts were screened for relevance. Full-text screening was then conducted for potentially eligible studies. Disagreements at each stage were resolved through consensus or consultation with an expert.

Data Extraction

A standardized data extraction sheet was developed and piloted. Extracted data included study characteristics (author, year, country, design, sample size), participant demographics, TMS protocol, comparator/control groups, primary and secondary outcomes, adverse events, dropout rates, and predictors of response. Data extraction was independently performed.

Risk of Bias Assessment

The Cochrane Risk of Bias tool 2.0 (RoB 2) [9] was used for assessing RCTs. For non-randomized studies, the ROBINS-I tool [10] was applied. Each study was independently evaluated by the author, and discrepancies were resolved through consensus. The overall risk of bias was categorized as low, some concerns, or high.

Data Synthesis

Due to substantial heterogeneity across the included studies in terms of TMS protocols (stimulation parameters, target brain regions, session frequency), study designs (RCTs, open-label, pilot studies), and outcome measures (different depression and OCD scales), a quantitative synthesis through meta-analysis was deemed inappropriate. Instead, findings were synthesized narratively, structured around three major domains: efficacy, safety and tolerability, and predictors of treatment response.

Results

Study Selection Results

**Figure 1:**
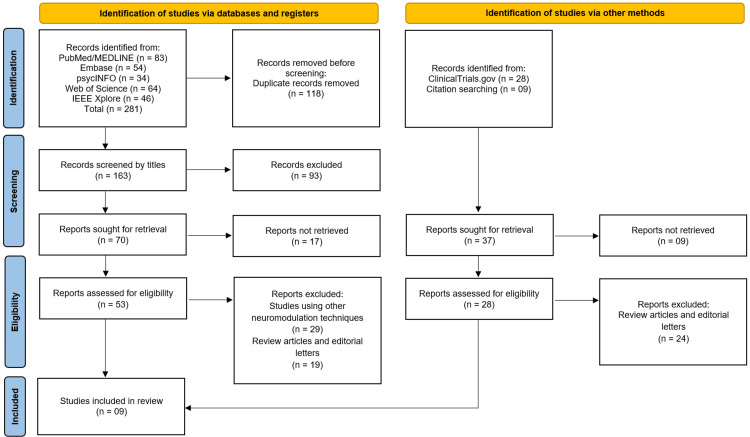
Studies Selection Process Illustrated on PRISMA Flowchart PRISMA: Preferred Reporting Items for Systematic Reviews and Meta-Analyses

The study selection process followed the PRISMA guidelines and is summarized in the attached flow diagram. A total of 281 records were identified through database searches, including PubMed/MEDLINE (n = 83), Embase (n = 54), PsycINFO (n = 34), Web of Science (n = 64), and IEEE Xplore (n = 46). An additional 37 records were identified through other sources, including ClinicalTrials.gov (n = 28) and citation searching (n = 9). After removal of 118 duplicates, 163 records were screened by title, of which 93 were excluded. Seventy full-text reports were sought for retrieval, with 17 not retrieved, leaving 53 reports for eligibility assessment. Of these, 29 were excluded for using other neuromodulation techniques, and 19 were excluded as review articles or editorial letters. From other sources, 28 reports were assessed for eligibility, with 24 excluded as review articles or editorial letters. This process culminated in the inclusion of nine studies [11-19] that met all predefined criteria for this systematic review (Figure 1).

Study Characteristics

A total of nine studies [11-19] were included in this systematic review, comprising six studies on OCD [11-16] and three studies on depressive disorders in adolescent populations [17-19]. The key characteristics of these studies are summarized in Table 3. The study designs were predominantly RCTs [11-16,19], with two open-label pilot studies focusing on adolescents with treatment-resistant depression (TRD) [17,18]. Sample sizes were generally small, ranging from 10 to 45 participants in the RCTs and 10 to 15 in the pilot studies. The populations included adults with OCD [11-16] and adolescents with either TRD [17,18] or first-episode depression [19].

**Table 3:** Characteristics of the Included Studies TMS: transcranial magnetic stimulation; rTMS: repetitive transcranial magnetic stimulation; OCD: obsessive-compulsive disorder; RCTs: randomized controlled trials; tDCS: transcranial direct current stimulation; NR: not reported; Y-BOCS: Yale-Brown Obsessive-Compulsive Scale; HAM-A: Hamilton Anxiety Rating Scale; HAM-D: Hamilton Depression Rating Scale; CGI-S: Clinical Global Illness - Severity; TBS: theta-burst stimulation; dACC: dorsal anterior cingulate cortex

First Author (Year)	Country	Study Design	Sample Size (n)	Population (Diagnosis)	Age Range/Mean Age	TMS Protocol (Type, Target, Frequency, Sessions)	Comparator/Control	Primary Outcomes	Follow-Up Duration
Dutta et al., [11] (2021)	India	RCT	33 (Active cTBS: 18; Sham: 15)	OCD	Adults	cTBS targeting OFC; intensive schedule: 10 sessions total (2/day for 5 days; 1 week), 1,200 pulses total across sessions	Sham stimulation	Y-BOCS, HAM-A, HAM-D, CGI-S. Group×time effects were significant for Y-BOCS, HAM-A, HAM-D, and CGI-S; after controlling for confounders, only HAM-A and CGI-S remained significant.	Assessments at the end of treatment and 2 weeks post-TBS
Meek et al., [12] (2021)	Canada	RCT	20 (Active = 10; Sham = 10)	OCD Patients	Adults	rTMS; target: dACC; frequency: NR (low-frequency stated); sessions: 20; schedule: twice daily	Sham rTMS	Error-monitoring performance; OCD symptom severity (Y-BOCS)	Symptom follow-up mentioned at 1 month and at 3 months
Liu et al., [13] (2021)	China	RCT	28	Treatment-resistant OCD participants	Adults	cTBS to the right OFC. Twice daily, 5 days/week for 2 weeks. (Total sessions: 2 sessions/day × 5 days/week × 2 weeks = 20 sessions.)	Sham cTBS (double-blinded)	Primary: Y-BOCS—clinical response. Secondary: anxiety symptoms, responder rates, depressive symptoms (noted improvement in depressive symptoms in the active group at 2 weeks).	Outcomes reported at 2 weeks; additional assessment at 6 weeks—so at least 6 weeks of follow-up reported.
Mukherjee et al., [14] (2023)	India	RCT	Enrolled n = 32 (completed n = 26)	Patients with OCD	Adults	Accelerated robotized neuronavigated cTBS over SMA. Bursts of 3 stimuli at 50 Hz, at 80% of MT, repeated at 5 Hz. 2 sessions/day × 900 pulses each; 30 sessions total over 3 weeks	Sham cTBS	Y-BOCS: obsession, compulsion, total, CGI-S, CGI-C, HAM-D, HAM-A	Outcomes assessed at the end of week 3 and week 8 (last follow-up = 8 weeks)
Ozer et al., [15] (2024)	Turkey	Parallel-group (active vs sham/placebo); randomization	Active = 14; Sham = 15 (Total = 29)	Treatment-resistant OCD patients	Adults	Double-cone coil dTMS targeting mPFC and ACC; 20 Hz; twice daily for 3 weeks	Placebo coil (sham TMS); concurrent antidepressant/antipsychotic meds continued at the same dose.	Primary reported outcomes: change in Y-BOCS scores (significantly greater reduction in active vs sham); change in HAM-A (greater reduction in active vs sham). Symmetry-related OCD symptoms: no significant difference.	NR
Jiang et al., [16] (2023)	China	RCT	45	OCD patients	Adults	ahTBS: accelerated high-dose theta burst stimulation; 1-Hz rTMS: traditional repetitive TMS at 1 Hz; Target not reported; Sessions: daily treatment for 5 days.	1-Hz rTMS (active comparator)	Change in Y-BOCS score (primary reported); also depression & anxiety measures and response rate (secondary).	Immediate post-treatment (end of 5 days).
Thai et al., [17] (2024)	USA	Pilot, open-label (single-arm)	15 (14 completed)	Adolescents with TRD	Mean 16.4 years (SD 1.42)	Deep TMS targeting the left dorsolateral prefrontal cortex (L-DLPFC). 10 Hz, 3.6 s train duration, 20 s inter-train interval, 55 trains → 1980 pulses/session; intensity 80–120% motor threshold; 30 sessions delivered daily over 6 weeks.	None (no sham/no active comparator)	Depression symptom severity measured by CDRS-R; also, safety, tolerability, and neurocognitive assessments	Pre- to immediate post-treatment (assessments before and after 6-week course).
Nair et al., [18] (2025)	USA	Open-label pre-post intervention study	10 (behavioral), 9 (fMRI)	Adolescents with TRD	Mean = 16.3 years (SD = 1.09)	Deep TMS (dTMS), left dlPFC, 30 sessions; frequency/intensity not reported	None (no sham/control group)	Affective bias (Word-Face Stroop), neural activation (fMRI amygdala and dlPFC), and correlation with clinical improvement	Immediate post-treatment
Jiao et al., [19] (2024)	China	Randomized (patients randomly assigned to sham, low-rTMS, or high-rTMS groups)	135	First-episode adolescent depression	NR	Low rTMS group and high rTMS group. The abstract does not provide details on stimulation type, cortical target, frequency (Hz), intensity, or total number of sessions.	Sham rTMS + fluoxetine	Depression severity (HAMD-17 change; response/effectiveness rates), CGI-I, cognitive function (WCST: erroneous responses, persistent errors, categorizations), adverse reactions	Outcomes reported at 2 weeks and 4 weeks (primary reported follow-up ≤ 4 weeks)

The TMS protocols were highly heterogeneous. For OCD, targets included the orbitofrontal cortex (OFC) [11,13], dorsal anterior cingulate cortex (dACC) [12], SMA [14], and medial prefrontal cortex/anterior cingulate cortex (mPFC/ACC) using a deep TMS (dTMS) double-cone coil [15]. Protocols varied from standard once-daily applications to accelerated, intensive schedules involving multiple daily sessions over a shortened treatment course (e.g., two to three weeks). Theta-burst stimulation (TBS), a patterned form of TMS, was used in four studies [11,13,15,16]. For adolescent depression, all studies targeted the left dorsolateral prefrontal cortex (dlPFC) [17-19], with two utilizing deep TMS systems [17,18]. Comparator groups consisted primarily of sham stimulation [11-16,19], though one depression study used an active comparator (1 Hz rTMS) [16] and two had no control group [17,18]. The primary outcome measures were the Yale-Brown Obsessive-Compulsive Scale (Y-BOCS) for OCD studies and either the Children's Depression Rating Scale-Revised (CDRS-R) [17] or the Hamilton Depression Rating Scale (HAMD-17) [19] for depression studies. Follow-up duration also varied significantly, from immediate post-treatment assessment to three months.

Efficacy of TMS

The efficacy outcomes, detailed in Table 4, were mixed but generally indicated positive effects for both disorders.

**Table 4:** Summary of Findings: Efficacy, Safety, and Predictors of Response TMS: transcranial magnetic stimulation; rTMS: repetitive transcranial magnetic stimulation; OCD: obsessive-compulsive disorder; RCTs: randomized controlled trials; NR: not reported; Y-BOCS: Yale-Brown Obsessive-Compulsive Scale; HAM-A: Hamilton Anxiety Rating Scale; HAM-D: Hamilton Depression Rating Scale; CGI-S: Clinical Global Illness - Severity; TBS: theta-burst stimulation; dACC: dorsal anterior cingulate cortex; SMA: supplementary motor area

First Author (Year)	Efficacy Outcomes (Response/Remission Rates, Symptom Reduction)	Safety & Tolerability (Adverse Events, Dropout)	Predictors of Treatment Response (Clinical, Demographic, Neurobiological)	Key Conclusions
Dutta et al., [11] (2021)	Symptom reduction: Repeated-measures ANOVA showed a significant group × time effect (pre → 2 weeks post-TBS) for Y-BOCS obsessions and compulsions, HAM-A, HAM-D, and CGI-S. After controlling for confounders, only HAM-A (anxiety) and CGI effects remained significant. Response/remission rates: Follow-up assessments at the end of the last session and 2 weeks post-TBS.	Described as well-tolerated	NR	iOFC-TBS vs. sham in OCD (n=33; active=18, sham=15; 10 sessions over 5 days, 2/day; total 1200 pulses/day). Short-term improvement primarily in anxiety symptoms (HAM-A) and global severity (CGI) after adjusting for confounders; suggests modulation of state-dependent dysregulation. Overall, well-tolerated.
Meek et al., [12] (2021)	Symptom reduction: Active LF-rTMS to dACC showed a 28.0% Y-BOCS reduction at 1 month vs. 11.7% with sham. Improved error-monitoring performance. Follow-up: symptoms assessed up to 3 months	Randomized, sham-controlled, double-blind; n=20, Active n=10, Sham n=10.	Implied neurocognitive link: Improvement in error-monitoring (dACC-related) co-occurred with clinical improvement, suggesting that error-monitoring impairment may be a neurobiological/cognitive marker related to treatment response.	LF rTMS targeting dACC can improve online behavioral adjustment and reduce OCD symptoms, supporting a mechanistic link between dACC hyperactivity/error-monitoring deficits and OCD pathophysiology.
Liu et al., [13] (2021)	Double-blind RCT; 28 treatment-resistant OCD participants; right OFC cTBS vs. sham; twice daily, 5 days/week for 2 weeks	No significant difference between active and sham on primary outcome (Y-BOCS) after 2 weeks (group×time F₂,₂₀=0.996, p=0.387). Secondary outcomes (anxiety, responder rates) also showed no significant group differences. Depressive symptoms improved in the active group at the end of treatment (p=0.027), but this difference was not significant at 6-week follow-up (p=0.089).	Reported as safe to use cTBS.	No clinical, demographic, or neurobiological predictors of response were described.
Mukherjee et al., [14] (2023)	Response/remission not reported; significant reduction in OCD symptoms (Y-BOCS obsession P<0.001 compulsion p=0.004, total illness severity cgi-c and depression in active ctbs vs. sham anxiety outcomes not significant)	Adverse events were not reported; the dropout rate was 18.8% (6/32).	No predictors assessed.	Adjunctive accelerated cTBS over SMA significantly improves OCD psychopathology, severity, and depression vs. sham; larger studies are needed.
Ozer et al., [15] (2024)	Significant reduction in Y-BOCS (25.36 ± 5.4 → 18.43 ± 6.86) and HAM-A (10.6 ± 3.5 → 6.7 ± 2.7) in active vs. sham group	No adverse events or dropout data reported; patients continued psychotropic meds at the same dose	No formal predictors assessed; symmetry-related OCD symptoms did not improve, suggesting a limited effect on this subtype	Adjunctive accelerated dTMS (double-cone coil, mPFC/ACC, 20 Hz, twice daily × 3 weeks) effective for treatment-resistant OCD, but safety data, remission rates, and predictors remain unclear
Jiang et al., [16] (2023)	Randomized trial; n = 45 OCD patients; ahTBS (accelerated high-dose theta burst stimulation) vs. 1-Hz rTMS; 5-day course; outcome = Y-BOCS; NCT05221632.	Both groups showed significant Y-BOCS reduction after 5 days (p < 0.001). No significant group × time interaction (F = 1.90, p = 0.18). No statistically significant difference in response/remission rates was reported; the authors note a trend toward higher response in the ahTBS group. Secondary outcomes (depression, anxiety) showed no between-group differences.	No negative cognitive side effects on neuropsychological testing for either treatment.	NR
Thai et al., [17] (2024)	Pilot open-label dTMS in adolescent TRD: N=15 (M age = 16.4 ± 1.42), 30 sessions, L-DLPFC, 10 Hz (BrainsWay H1 coil)	Response: 6/15 met criteria for treatment response (40%). Remission: not reported. Symptom reduction: CDRS-R severity significantly improved pre→post; clinically significant improvement noted after ~10 sessions.	Completion/dropout: 14/15 completed (1 discontinued). Adverse events: 1 participant experienced a convulsive syncope; the remainder had only mild side effects (e.g., headaches). The authors report no serious adverse events and minimal/no change in cognitive performance.	NR
Nair et al., [18] (2025)	Clinical improvement observed. Decreased neural activation correlated with clinical improvement	NR	Neurobiological: decreased amygdala activation (negative word/happy face) and reduced activation during congruent conditions correlated with clinical improvement	Preliminary evidence that dTMS targeting left dlPFC may normalize neural efficiency and affective bias in adolescents with TRD, though limited by small sample size, lack of sham control, and absent safety data
Jiao et al., [19] (2024)	Response rates: Low rTMS 95.6%, High rTMS 97.8% vs. Sham 80%; greater HAMD-17 and CGI-I reductions; improved WCST cognitive outcomes	NR	NR	rTMS + fluoxetine significantly enhanced antidepressant efficacy and cognitive function in first-episode adolescent depression with good tolerability

In OCD, four of the six RCTs reported statistically significant reductions in Y-BOCS scores for active TMS compared to sham stimulation [11,12,14,15]. Dutta et al. [11] found a significant group-by-time interaction for Y-BOCS, though after controlling for confounders, significant effects remained only for anxiety (HAM-A) and global severity (CGI-S). Meek et al. [12] demonstrated a 28% Y-BOCS reduction with active low-frequency rTMS to the dACC versus 11.7% with sham at one-month follow-up. Mukherjee et al. [14] and Ozer et al. [15] both reported significant improvements in Y-BOCS and other secondary measures like depression for their active accelerated TMS protocols. In contrast, two studies found no significant between-group differences for the primary Y-BOCS outcome. Liu et al. [13] reported no clinical benefit for right OFC cTBS over sham at two weeks, and Jiang et al. [16] found no statistically significant difference in Y-BOCS reduction between accelerated high-dose TBS and 1Hz rTMS after a five-day course, though a trend favored the TBS group.

In adolescent depression, all three studies reported symptom improvement. The open-label dTMS studies by Thai et al. [17] and Nair et al. [18] showed significant pre-to-post reduction in depression symptoms and observed clinical improvement correlated with neural changes, though the lack of a control group limits efficacy conclusions. The RCT by Jiao et al. [19] provided the strongest evidence, showing that both high- and low-intensity rTMS combined with fluoxetine resulted in significantly higher response rates (95.6% and 97.8%, respectively) and greater reduction in HAMD-17 scores compared to sham rTMS with fluoxetine (80%).

Safety and Tolerability

TMS was generally reported to be safe and well-tolerated across most studies. In the OCD RCTs, adverse events were rarely severe, and studies reported no significant differences in tolerability between active and sham groups [11-16]. Dropout rates were provided in some studies and were generally low or comparable between groups; for example, Mukherjee et al. [14] had an overall dropout rate of 18.8%.

In the adolescent depression studies, safety was a primary focus. Thai et al. [17] reported that dTMS was well-tolerated, with most side effects being mild (e.g., headaches). However, one serious adverse event was reported: a participant experienced a convulsive syncope, which resolved without sequelae; the authors noted that no clear causal factor related to the stimulation protocol was identified. The other studies [18,19] did not report any serious adverse events, with Jiao et al. [19] noting good tolerability for rTMS combined with medication. Neuropsychological testing in two studies [16,17] showed no negative cognitive side effects.

Predictors of Treatment Response

The investigation of predictors of treatment response was notably limited across the included studies. The majority did not formally assess or report on clinical, demographic, or neurobiological factors predictive of a positive outcome [11,13-17,19].

One exception was Meek et al. [12], who implied a neurocognitive predictor, finding that improvement in error-monitoring performance (a function linked to the dACC) co-occurred with clinical improvement in OCD symptoms. Another was Nair et al. [18], who identified a neural correlate of response, demonstrating that clinical improvement in adolescents with TRD was correlated with decreased amygdala activation and reduced dlPFC activation during an affective task. Ozer et al. [15] provided a clinical observation that symmetry-related OCD symptoms did not improve with dTMS, suggesting symptom subtype may predict a lack of response for certain protocols. Overall, the evidence for reliable predictors remains sparse and preliminary.

Risk of Bias Findings

Based on the risk of bias assessment, the overall methodological quality of the included studies was high for the majority of the RCTs. Six of the seven RCTs were judged to have a low overall risk of bias [11-13,15,16,19], indicating robust methodologies concerning randomization, outcome measurement, and reporting. One RCT was assessed as having a high overall risk of bias [14] due to some concerns regarding deviations from intended interventions and a high rate of missing outcome data. For the non-randomized studies, one open-label trial was judged to have a low overall risk of bias [17], while the other was judged to have a critical overall risk of bias [18], primarily due to a critical level of confounding and serious bias in the selection of participants (Tables 5, 6).

**Table 5:** Risk of Bias in Randomized Controlled Trials (Assessed With the Cochrane RoB 2 Tool)

Study (First Author, Year)	D1: Randomization Process	D2: Deviations From Intended Interventions	D3: Missing Outcome Data	D4: Measurement of the Outcome	D5: Selection of the Reported Result	Overall Risk of Bias
Dutta et al., [11] (2021)	Low	Low	Low	Low	Low	Low
Meek et al., [12] (2021)	Low	Low	Low	Low	Low	Low
Liu et al., [13] (2021)	Low	Low	Low	Low	Low	Low
Mukherjee et al., [14] (2023)	Low	Some Concerns	High	Low	Low	High
Ozer et al., [15] (2024)	Low	Low	Low	Low	Low	Low
Jiang et al., [16] (2023)	Low	Low	Low	Low	Low	Low
Jiao et al., [19] (2024)	Low	Low	Low	Low	Low	Low

**Table 6:** Risk of Bias in Non-Randomized Studies (Assessed With the ROBINS-I Tool)

Study (First Author, Year)	D1: Confounding	D2: Selection of Participants	D3: Classification of Interventions	D4: Deviations From Intended Interventions	D5: Missing Data	D6: Measurement of Outcomes	D7: Selection of Reported Result	Overall Risk of Bias
Thai et al., [17] (2024)	Low	Low	Low	Low	Low	Low	Low	Low
Nair et al., [18] (2025)	Critical	Serious	Moderate	Low	Low	Low	Low	Critical

Discussion

This systematic review evaluated the efficacy, safety, and predictors of treatment response for TMS in OCD and adolescent depression by synthesizing evidence from nine studies. The findings present a nuanced picture: TMS emerges as a promising therapeutic intervention with a generally favorable safety profile, but its application is marked by significant heterogeneity in protocols, variable efficacy outcomes, and a critical lack of data on predictors of response. The results in OCD are particularly intriguing, demonstrating that neuromodulation of various nodes within the CSTC (cortico-striato-thalamo-cortical) circuit can yield clinically meaningful improvements. The positive outcomes from studies targeting the dACC [12], OFC [11], SMA [14], and mPFC/ACC [15] reinforce the neurocircuitry model of OCD, suggesting that aberrant activity in this network is a viable therapeutic target.

The study by Meek et al. [12] is especially compelling, as it moves beyond mere symptom reduction to demonstrate a mechanistic link. The co-occurrence of improved error monitoring, a core neurocognitive deficit in OCD, with clinical improvement provides a plausible biological pathway for the therapeutic effect of dACC-targeted low-frequency rTMS. This finding aligns with a growing body of literature that emphasizes the importance of targeting specific cognitive functions through neuromodulation, rather than applying a one-size-fits-all approach to brain stimulation [20]. However, the inconsistent results cannot be ignored. The null findings from Liu et al. [13], who applied cTBS to the right OFC, and Jiang et al. [16], who found no difference between the two active protocols, underscore the profound impact of technical parameters such as target location, laterality, stimulation pattern, and dose. This heterogeneity mirrors the broader landscape of TMS for OCD, where meta-analyses confirm overall efficacy but report considerable variability, often attributed to differences in coil design, neuronavigation use, and patient resistance level [20]. The accelerated, intensive protocols tested in several studies [14,15] represent a significant shift from traditional daily treatment schedules, offering the potential for more rapid symptom relief. While the results are encouraging, the long-term durability of these effects remains an open question, as most studies had limited follow-up periods. This is a critical gap, as the chronic, relapsing nature of OCD necessitates interventions with sustained benefits.

The evidence for adolescent depression, though more limited, introduces several provocative possibilities. The stellar results from the RCT by Jiao et al. [19] suggest a paradigm shift. The near-universal response rates when rTMS is combined with fluoxetine in first-episode patients position TMS not merely as a rescue therapy for treatment-resistant cases but as a potent augmenting strategy at the onset of illness. This has profound implications for clinical practice, potentially offering a way to improve remission rates and alter the long-term trajectory of major depressive disorder in youth [21]. The proposed mechanism by which TMS potentiates the neuroplastic effects of antidepressants is supported by preclinical studies showing that TMS can enhance serotonin transmission and promote synaptogenesis [21]. The open-label studies in treatment-resistant adolescents [17,18] provide preliminary support for the safety and potential efficacy of dTMS, a technology that allows for stimulation of deeper cortical structures. The correlation between clinical improvement and normalization of neural activation patterns, particularly decreased amygdala hyperactivity [18], is a crucial finding. It provides a neurobiological signature for treatment response, echoing extensive research in adults that links amygdala hyperactivity to negative affective bias and treatment resistance [22]. However, the uncontrolled design of these studies means that the observed effects could be influenced by placebo responses, regression to the mean, or the natural fluctuation of symptoms. The serious adverse event of convulsive syncope reported by Thai et al. [17] serves as a vital reminder that even non-invasive brain stimulation carries risks and must be administered in a controlled setting with appropriate medical oversight, especially in adolescent populations; however, the authors did not provide a specific rationale or mechanistic explanation for this event beyond noting it as an isolated occurrence.

A paramount finding of this review is the exceptional safety and tolerability profile of TMS across all studies. The predominance of mild, transient adverse effects, such as scalp discomfort and headache, is consistent with the extensive safety data from adult populations [23]. The absence of any significant negative impact on neurocognitive functioning [16,17] is perhaps the most reassuring finding for the application of TMS in adolescents, whose brains are still undergoing critical development. This safety profile starkly contrasts with that of other neuromodulation therapies like ECT, which, while effective, carries a higher burden of cognitive side effects and stigma [24]. This makes TMS a more palatable and accessible option for a wider range of patients, including those earlier in their illness course, and it is further supported by established safety guidelines for adults and emerging recommendations for adolescents, which emphasize precautions such as screening for epilepsy or seizure history, avoiding use in patients with implanted metallic or electronic devices, and ensuring treatment is delivered under trained medical supervision.

Perhaps the most significant gap identified is the stark absence of research into predictors of treatment response. The field seems stalled at demonstrating whether TMS works, neglecting the more clinically pertinent question of for whom it works best. The few glimpses provided, error-monitoring improvement [12], amygdala activation [18], and symptom subtype [15], are tantalizing but preliminary. They hint at a future where treatment selection is guided by cognitive profiling, neuroimaging biomarkers, and clinical characteristics, moving toward personalized neuromodulation. This gap is not unique to this review but reflects a broader shortcoming in the TMS literature. For instance, a large-scale study in adult depression found that older age, less treatment resistance, and certain neuroanatomical features were associated with better response to dlPFC TMS [25]. Similar large, multidisciplinary efforts are urgently needed in OCD and adolescent depression to identify robust predictors that can guide clinical decision-making and improve overall response rates.

The overall high methodological quality of the included RCTs, with six out of seven rated as low risk of bias, strengthens confidence in the positive efficacy results. However, the high risk of bias in one RCT [14] due to missing outcome data and the critical risk of bias in one open-label study [18] due to confounding highlight that methodological rigor remains variable. Future studies must prioritize complete outcome reporting and, where feasible, employ randomized controlled designs to minimize the potential for bias.

Limitations

This systematic review has several limitations that must be acknowledged. First, the small number of studies, particularly for adolescent depression (n=3), limits the robustness and generalizability of the conclusions. Second, the substantial clinical and methodological heterogeneity observed in populations (adults with OCD vs. adolescents with depression), TMS protocols (target, frequency, pattern, and number of sessions), and comparator groups precluded a quantitative meta-analysis, making it difficult to derive a pooled estimate of effect size. Third, the follow-up durations in most studies were short-term, leaving the critical question of the long-term durability of TMS effects unanswered. Fourth, the risk of bias was not low for all studies, with one RCT rated high risk and one non-randomized study rated critical risk, which could influence the overall interpretation of findings. Finally, the almost complete absence of data on predictors of response represents a fundamental knowledge gap that future research must address.

Study Strengths, Clinical Implications, and Future Directions

This systematic review has several strengths, including adherence to PRISMA guidelines, a comprehensive multi-database search strategy, and the inclusion of both RCTs and non-randomized interventional studies, which together provide a broad and balanced synthesis of current evidence. Clinically, the findings reinforce that TMS is generally safe and well-tolerated in adolescents, with promising efficacy in both depression and OCD, suggesting that it may serve as a viable alternative or augmentation strategy where pharmacological and psychotherapeutic treatments yield suboptimal results. Importantly, its favorable safety profile compared to other neuromodulation techniques highlights its potential for earlier intervention in the illness trajectory. Future research should focus on large-scale, multi-center RCTs with longer follow-up durations to assess the durability of treatment effects, standardized stimulation protocols to reduce heterogeneity, and integration of multimodal biomarkers, including neuroimaging, neurocognitive, and clinical predictors, to move toward precision medicine approaches in adolescent psychiatry.

## Conclusions

TMS is a promising and safe therapeutic modality for both OCD and adolescent depression. For OCD, evidence supports the efficacy of modulating various targets within the CSTC circuit, though the optimal parameters and long-term benefits require further definition. For adolescent depression, TMS shows potential both as a powerful augmenting strategy in first-episode patients and as an intervention for treatment-resistant cases, though more rigorous and larger RCTs are needed to confirm these preliminary findings. The consistently favorable safety profile across studies is a major asset for its clinical translation. However, the most critical conclusion is that the field must now pivot. Future research cannot simply continue to test efficacy in heterogeneous groups. It must be characterized by larger, well-designed, and collaborative studies that prioritize the identification of clinical, cognitive, and neurobiological predictors of response. The ultimate goal is to move beyond the question of "Does TMS work?" to answer "How can we make TMS work for this patient?" Achieving this will be essential for delivering on the promise of personalized and effective neuromodulation therapies for these debilitating disorders.
